# Comparing Mental Health of Athletes and Non-athletes as They Emerge From a COVID-19 Pandemic Lockdown

**DOI:** 10.3389/fspor.2021.612532

**Published:** 2021-05-20

**Authors:** Christopher Knowles, Stephen Shannon, Garry Prentice, Gavin Breslin

**Affiliations:** ^1^School of Sport, Ulster University, Belfast, United Kingdom; ^2^School of Sport, Ulster University, Londonderry, United Kingdom; ^3^Bamford Centre for Mental Health and Wellbeing, Ulster University, Coleraine, United Kingdom; ^4^School of Arts, Dublin Business School, Dublin, Ireland; ^5^School of Psychology, Ulster University, Coleraine, United Kingdom

**Keywords:** athlete, athletic identity, resilience, wellbeing, depression, anxiety, loneliness

## Abstract

Athletes going through transition periods such as injury or retirement have previously reported feelings of depression and anxiety, especially when feeling unsupported. Cessation of competitive sport during the pandemic has forced athletes through a non-normative transition and has reduced many opportunities to satisfy their basic psychological needs increasing the risk of poor wellbeing and loneliness. Whilst athletes are often praised for their resilience—a trait that serves to support them during tough times—the inability to play sport can be particularly challenging for those with strong athletic identities. An online cross-sectional survey (*n* = 744) was conducted to capture adult athlete and non-athlete mental health factors (specifically wellbeing, depression, anxiety, loneliness) during emergence from a COVID-19 lockdown. Results showed that resilience was positively correlated with mental health but was no higher in athletes than non-athletes. Furthermore, athletes reported greater anxiety than non-athletes, a difference mediated by negative affectivity—a subfactor of athletic identity. We present evidence that after a temporary transition away from sport, athletes' resilience is comparable to non-athletes leaving them just as likely to suffer poor mental health. Moreover, athletes with strong athletic identities are likely to experience anxiety symptoms above and beyond those reported by non-athletes. Findings have implications for the development of self-management guidance for athletes as the COVID-19 pandemic and restrictions on sport participation continue.

## Introduction

Mental health is defined by the World Health Organisation (WHO) as, “a state of well-being in which an individual realises his or her own abilities, can cope with the normal stresses of life, can work productively, and is able to make a contribution to his or her community” (World Health Organisation, 2018). In a time when the world is on high alert due to the uncertainty and distress caused by a global pandemic, research into mental health and self-management has become increasingly important. Due to the rapid spread of COVID-19, the United Kingdom's government announced a national lockdown on 23rd March 2020 signifying a major step in a monumental transition from normal daily living to a world where mask wearing, social distancing and perpetual uncertainty have become the norm. A significant feature of “the COVID experience” (Samuel et al., 2020) has been the abrupt and repeated halting of competitive sport due to national lockdowns or outbreaks of the virus in sport teams and/or their respective institutions. The stop-start nature of many leagues and competitions that has existed over the course of the pandemic has presented a multitude of stressors detrimental not only to the personal lives of both amateur and professional athletes, but also to the development of their sporting careers.

The pandemic has constituted a long and enduring transitionary period in the life and career of an athlete (Stambulova et al., 2020). Transitions constitute critical phases for athletic development wherein stressors must be responded to positively to facilitate continued success, both within and out with sport (Stambulova, 2017; Stambulova et al., 2020). The predictability of a transition period can determine how successfully an athlete adapts and responds to stressors during this time with normative transitions typically being associated with more positive mental health outcomes than non-normative transitions (Wheaton, 1990; Stambulova, 2003, 2017). The most commonly observed mental health outcomes associated with non-normative transitions in sport (e.g., injury, de-selection, premature retirement) include feelings of depression and anxiety (Wylleman et al., 2004; Appaneal et al., 2009; Rice et al., 2016). Due to its non-normative nature, it was expected that similar outcomes would arise due to COVID-19. Moreover, during the pandemic athletes have had fewer opportunities to meet their basic psychological needs (Ryan and Deci, 2017) and/or benefit from sport-related social support networks (Ntoumanis et al., 2009; Biddle et al., 2015) which can contribute to poor wellbeing and loneliness (Lippke et al., 2021).

Often cited as a key determinant of an individual's mental health when faced with adversity is their level of resilience (Fletcher and Sarkar, 2013, 2016). To this end, it is possible that athletes may have an advantage over non-athletes given sports' long-standing association with the development of this beneficial psychological trait (Caddick and Ryall, 2012). Some studies support this notion finding athletes to be more resilient than non-athletes (Guillén and Laborde, 2014; Laborde et al., 2016). Considering this, it could be argued that athletes may be better equipped to deal with the psychological burden of the pandemic than non-athletes as a result of their conditioning to thrive in challenging environments and competition. That being said, existing resilience studies have investigated samples of athletes regularly participating in sport and so findings may not be representative of those undergoing a period of transition. Therefore, there is a need for further exploratory research in this area.

Another key determinant of athlete mental health during transitions is often their athletic identity (Ronkainen et al., 2016). Athletic identity can be understood as a multidimensional construct within the self-concept comprising of self-related information garnered from psychosocial factors that accompany the athlete role (Brewer et al., 1993; Murphy et al., 1996; Ronkainen et al., 2016). Brewer and colleagues propose a three-factor model for understanding athletic identity. *Social identity* considers the extent to which an individual identifies themselves as an athlete; *exclusivity* considers the degree to which the individual's self-worth depends on their athletic identity in comparison to other roles they fulfil (e.g., parent, student); and *negative affectivity* assesses the negative emotions that occur due to non-participation (Brewer et al., 1993). For some athletes, extensive involvement in sport over time can cause the athlete role to become deeply entwinned in their personal identity, engulfing their sense-of-self, social life and/or career. These individuals are often the most at risk for suffering poor mental health when going through a transition at which time the opportunity to participate in sport is unavailable (Grove et al., 1997; Alfermann et al., 2004; Wylleman et al., 2004; Sanders and Stevinson, 2017).

The aim of the current study was to investigate the relationships between resilience, athlete identity and mental health during transition periods. Specifically, the study aimed to identify whether associations existed between athlete identity, resilience, wellbeing, anxiety, depression and/or loneliness as we emerged from a national lockdown to better understand the impact of the pandemic. Hypothesis one was that resilience would be positively correlated with mental health. Hypothesis two was that athlete identity would be negatively correlated with mental health. Hypothesis three was that resilience would be higher in athletes than non-athletes. As this is an exploratory piece of research, in the event we came across unexpected results, we planned to conduct mediation and/or moderation analysis to ascertain why our findings might have occurred.

## Method

### Recruitment and Participants

**Figure 1:**
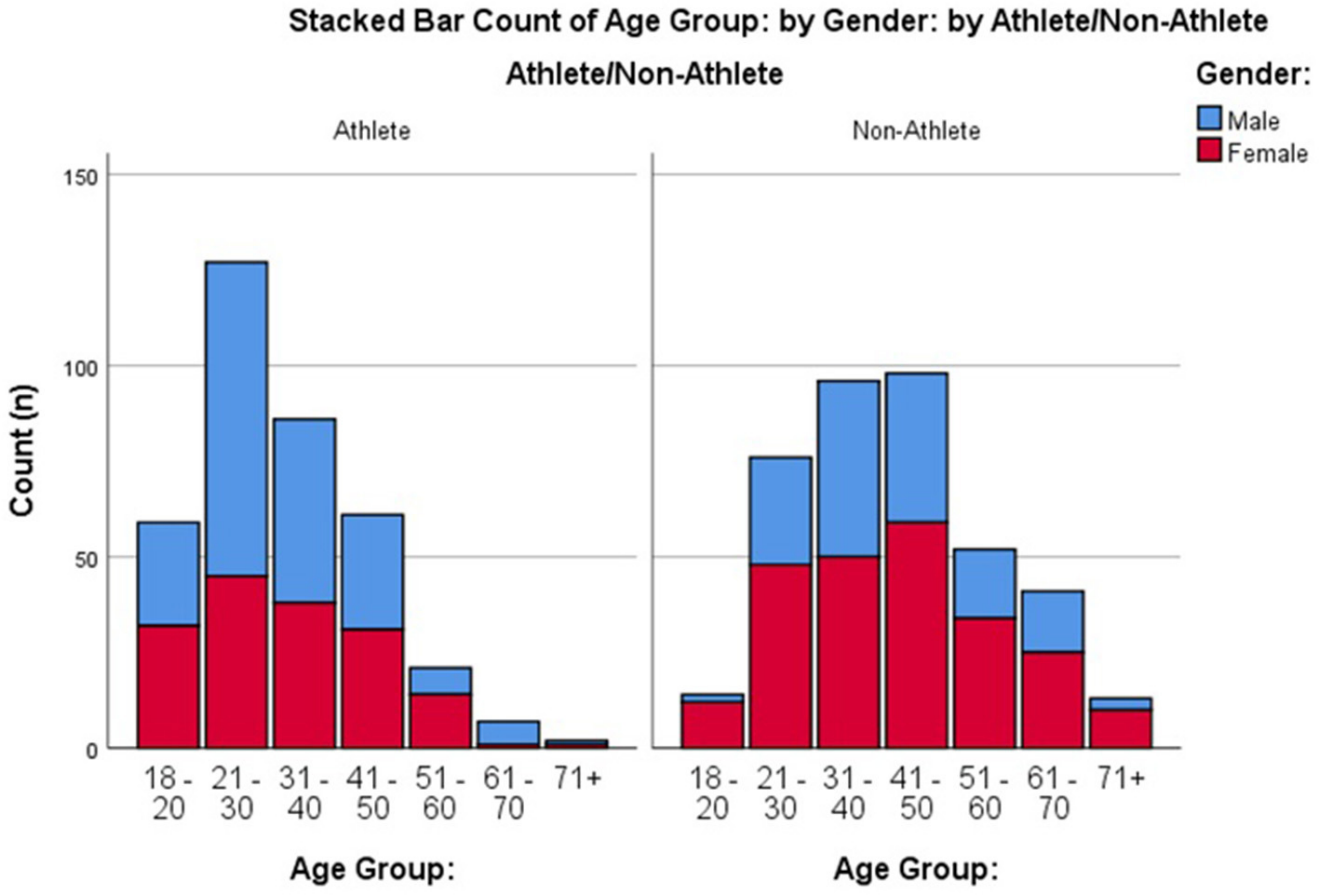
Histogram detailing the gender split and age ranges for athlete and non-athlete groups.

**Figure 2:**
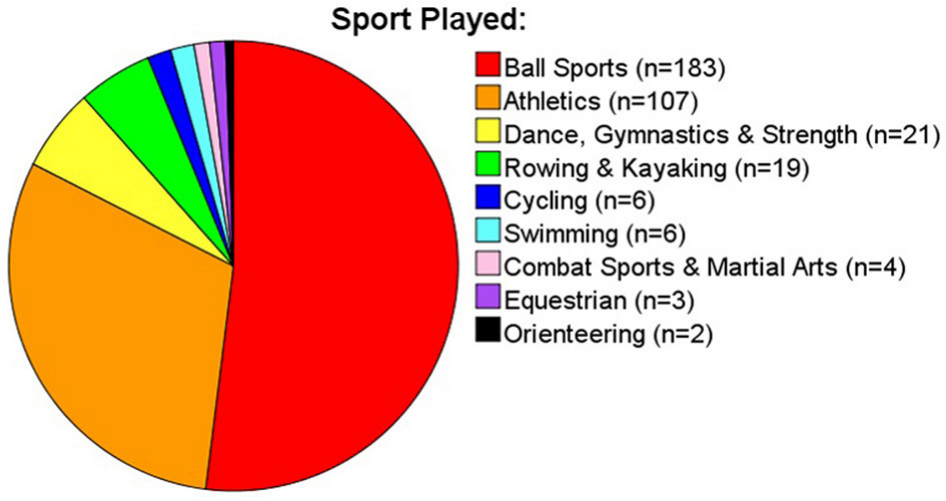
Pie chart of sports played by the athlete sample.

**Figure 3:**
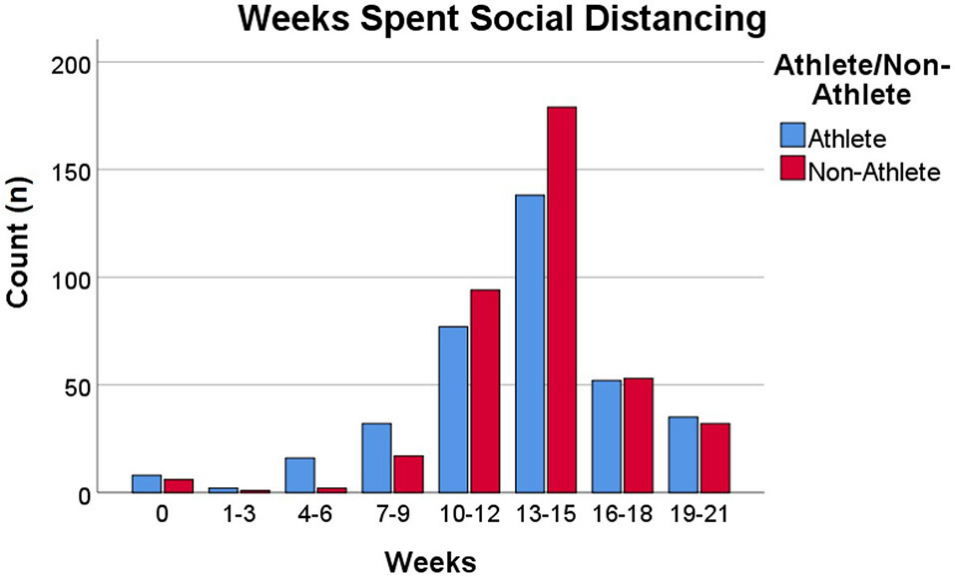
Weeks spent social distancing by both athletes and non-athletes.

Ethical approval for the study was granted by Ulster University. All participants provided informed consent and were free to withdraw at any time. No personal identifying data was collected to ensure confidentiality. A cross-sectional survey research design was used. Data was collected between 23/06/2020-−13/07/2020 (i.e., 14–16 weeks from the start of lockdown, 2 weeks after restrictions were lifted for those shielding in their homes). All data was collected via SurveyMonkey. A total of 20 sporting organisations and governing bodies of sport from across the United Kingdom and Republic of Ireland were contacted via email to take part. Sporting associations publicly shared a link to the survey via their Twitter page, website and/or members mailing lists. Non-athlete participants were recruited by word of mouth and via an article published in a Northern Ireland online newspaper. A total of 753 participants over the age of 18 were recruited. Of these, nine respondents resided outside the United Kingdom or Ireland during lockdown thus, were not considered in final analysis. This left 744 participants over the age of 18 to be included of which 558 lived in the United Kingdom and 186 in the Republic of Ireland (male athletes = 199, female athletes = 161, male non-athletes = 148, female non-athletes = 236). Descriptive data for the gender and age split of the sample is provided in Figure 1. Of the 360 athletes recruited, 351 reported their level of participation (243 non-elite; 64 semi-elite; 44 elite) and which sport they primarily played (see Figure 2). When the survey was conducted, 38% of athletes and 47% of non-athletes had been practising social distancing for 13–15 weeks all of which, except for 1 or 2 weeks depending on their response date, was spent in lockdown (described in greater detail in Figure 3). A roughly equal proportion of athletes (8%) and non-athletes (10%) had been shielding since the beginning of the pandemic. Participants also reported the size of their lockdown bubbles. Bubbles comprised of people in the immediate household plus all those from another household with whom they were allowed to mix with whilst social distancing. The size of participants' lockdown bubbles were largely consistent across both athletes (*M* = 3.52, *SD* = 1.44) and non-athletes (*M* = 3.20, *SD* = 1.40).

### Measures

All participants reported their gender, age range, occupation, marital status, smoking status, diet, number of weeks spent social distancing and the size of their social isolation bubble. The following question, taken from an operational definition of sport (Rejeski and Brawley, 1988; Shannon et al., 2019) was used to categorise participants as either athletes or non-athletes: “Are you an athlete involved in a structured, rule-bound, competitive physical activity?” The term “mental health” was used throughout analysis and refers collectively to participants' self-reports of wellbeing, anxiety, depression, and loneliness. Resilience and athletic identity were also surveyed via self-report measures. The following questionnaires were presented:

#### The Adult Mental Health Continuum–Short Form

The MHC-SF (Keyes, 2005) is a validated three factor 14-item questionnaire measuring wellbeing. The MHC-SF provides a score for overall wellbeing and individual scores for three subscales: social, emotional, and psychological wellbeing. Using a 7-point Likert scale, the summed score for overall wellbeing and each sub factor was calculated to produce four individual variables. The MHC-SF also includes a measure of whether participants are flourishing, languishing or are moderately mentally healthy (for thresholds see Keyes, 2009). The scale had high internal consistency (Cronbach's α = 0.93) as do the social (α = 0.84), emotional (α = 0.87), and psychological (α = 0.88) subscales.

#### The Hospital Anxiety and Depression Scale

The HADS (Zigmond and Snaith, 1983) is a two-factor 14-item scale used to rate the severity of anxiety and depressive symptoms. Responses were recorded on a Likert scale ranging from 0 to 3 with the sum of participant responses providing individual scores for anxiety (HADS-A) and depression (HADS-D). Scores of 0–7 are considered within the normal range, 8–10 are evidence of mild symptoms, and 11–21 are suggestive of moderate/severe symptoms (Snaith, 2003; Breeman et al., 2015). Cronbach's α was 0.89 for the scale overall, 0.86 for HADS-A and 0.81 for the HADS-D subscales.

#### The Brief Resilience Scale

The BRS (Smith et al., 2008) contains 6-items that provide a single score for resilience measured on a 5-point Likert scale. The sum of participant responses gave their level of resilience. The scale had a high level of internal consistency (Cronbach's α = 0.89).

#### The Short Loneliness Scale

The SLS (De Jong Gierveld and Van Tilburg, 2006) is a two-factor scale containing 6-items measuring overall (Lone-O), social (Lone-S), and emotional loneliness (Lone-E). The measure has been validated cross-culturally and had an acceptable level of internal consistency in the current sample (α = 0.70). Scores were measured on a 5-point Likert scale. For negatively worded items (e.g., I often feel rejected), neutral and positive answers (i.e., more or less, agree, strongly agree) were counted. For positively worded items (e.g., there are enough people I feel close to) the neutral and negative answers were counted. A score of 0 infers complete social embeddedness whereas a score of six (or three for either subscale) is evidence of extreme loneliness.

#### The Athletic Identity Measurement Scale

The three factorial AIMS (Brewer et al., 1993), a 7-point Likert scale, is a measure of athletic identity in which three individual factors—social identity (AIMS-SI), negative affectivity (AIMS-NA), and exclusivity (AIMS-E)—are subordinate to one higher order factor—overall athletic identity (AIMS-O). Participants' summed scores offered a measure of their athletic identity. The AIMS had a high level of internal consistency (α = 0.89) as did each subscale: AIMS-SI (α = 0.84); AIMS-E (α = 0.86); and AIMS-NA (α = 0.78).

## Data Handling

The mean or sum of participants' responses were calculated as per the scoring criteria for each measure. Analysis was conducted using Statistical Package for the Social Sciences 26 (copyright IBM corp., NY, USA) with the alpha level set to *p* < 0.05. Pearson's correlations were considered weak, moderate and strong when *r* = 0.20, 0.50, and 0.80, respectively. Effect sizes for *t*-tests were interpreted using Cohen's d where small, medium and large effects were classed as 0.20, 0.50, and 0.80, respectively. Effect size for univariate analysis was interpreted using partial eta squared where small to large effect sizes = 0.01, 0.06, and 0.14, respectively (Field, 2013). Given the sample size was large (*N* = 744), central limit theorem inferred the data was normally distributed. Levene's tests inferred homogeneity of variances for all statistical tests henceforth. Mediation analysis was carried out in line with Newsom's (2020) method using a series of linear regressions and Sobel tests. An initial regression was conducted to compute the unstandardised coefficient for the relationship between the predictor variable and the mediator. A second regression was then conducted to obtain the unstandardised coefficient for the path between the mediator and the outcome variable when the predictors were also included in the model. Partial mediation was observed when Sobel tests returned a positive result but the relationship between a predictor and an outcome variable remained significant after controlling for the potential mediator. Full mediation occurred in instances where Sobel tests were positive but the relationship between a predictor and dependent variable became non-significant when controlling for the mediator (Newsom, 2020).

## Results

Table 1 describes the prevalence of anxiety and depressive symptoms, extreme forms of loneliness, flourishing, languishing and moderately mentally healthy athletes and non-athletes as per the criteria of the HADS, SLS, and MHC-SF, respectively.

**Table 1:** Descriptive statistics for the severity of anxiety and depressive symptoms, and the rate of flourishing, languishing, and extreme loneliness in athletes and non-athletes.

**Mental health**		**Athlete**		**Non-athlete**	
		***n***	**%**	***n***	**%**
Anxiety	Normal range	153	47.2	187	55.2
	Mild	83	25.6	65	19.2
	Moderate/Severe	88	27.2	87	25.7
Depression	Normal range	242	74.7	247	72.9
	Mild	59	18.2	61	18
	Moderate/Severe	23	7.1	31	9.1
Overall loneliness	No evidence of loneliness	9	2.8	16	4.9
	Extremely lonely	29	9.1	38	11.6
Emotional loneliness	No evidence of loneliness	24	7.6	27	8.2
	Extremely lonely	79	24.9	82	24.9
Social loneliness	No evidence of loneliness	135	42.6	141	42.9
	Extremely lonely	55	17.4	55	16.7
Wellbeing	Flourishing	32	9.7	45	12.9
	Moderately mentally healthy	290	87.9	290	83.1
	Languishing	8	2.4	14	4

### Testing Hypothesis 1: Resilience Will Be Positively Correlated With Mental Health

Pearson's product moment correlations were conducted for athletes and non-athletes to measure the relationship between resilience and mental health. Findings showed that resilience was moderately positively correlated with all four MHC-SF variables (i.e., overall, social, psychological, and emotional wellbeing) and moderately negatively correlated with all SLS variables, HADS-A and HADS-D. These relationships were observed in both athletes and non-athletes which supports our first hypothesis. See Tables 2, 3 for descriptive statistics.

**Table 2:** Descriptive statistics and correlations for athletes.

	**Athletes**	***n***	***M***	***SD***	**1**	**2**	**3**	**4**	**5**	**6**	**7**	**8**	**9**	**10**	**11**	**12**	**13**	**14**
1.	Resilience	319	21.32	4.77	-													
2.	AIMS: Overall	353	5.48	0.83	−0.103	-												
3.	Social identity	353	5.73	0.89	0.036	0.744^**^	-											
4.	Exclusivity	353	5.01	1.39	−0.093	0.821^**^	0.431^**^	-										
5.	Negative affectivity	353	5.58	1.16	−0.188^**^	0.658^**^	0.190^**^	0.355^**^	-									
6.	Overall wellbeing	330	43.31	13.13	0.479^**^	−0.044	0.162^**^	−0.086	−0.192^**^	-								
7.	Emotional wellbeing	330	10.65	2.79	0.468^**^	−0.115^*^	0.118^*^	−0.172^**^	−0.216^**^	0.789^**^	-							
8.	Social wellbeing	330	12.62	5.83	0.346^**^	0.013	0.154^**^	−0.030	−0.107	0.884^**^	0.611^**^	-						
9.	Psychological wellbeing	330	20.03	6.40	0.461^**^	−0.052	0.140^*^	−0.075	−0.202^**^	0.902^**^	0.625^**^	0.635^**^	-					
10	Anxiety	324	8.09	4.31	−0.558^**^	0.166^**^	−0.007	0.158^**^	0.234^**^	−0.555^**^	−0.578^**^	−0.448^**^	−0.477^**^	-				
11.	Depression	324	5.12	3.59	−0.520^**^	0.075	−0.127^*^	0.116^*^	0.195^**^	−0.661^**^	−0.653^**^	−0.532^**^	−0.584^**^	0.645^**^	-			
12.	Overall loneliness	317	2.66	0.71	−0.478^**^	0.163^**^	−0.059	0.161^**^	0.280^**^	−0.681^**^	−0.614^**^	−0.556^**^	−0.620^**^	0.560^**^	0.635^**^	-		
13.	Emotional loneliness	317	2.96	0.79	−0.442^**^	0.146^**^	−0.048	0.134^*^	0.258^**^	−0.597^**^	−0.605^**^	−0.479^**^	−0.524^**^	0.576^**^	0.588^**^	0.814^**^	-	
14.	Social loneliness	317	2.36	0.90	−0.366^**^	0.129^*^	−0.050	0.136^*^	0.216^**^	−0.549^**^	−0.437^**^	−0.457^**^	−0.518^**^	0.377^**^	0.485^**^	0.861^**^	0.406^**^	-

* *Correlation significant at the 0.05 level (2-tailed)*.

** *Correlation significant at the 0.01 level (2-tailed)*.

**Table 3:** Descriptive statistics and correlations for non-athletes.

	**Non-athletes**	***n***	***M***	***SD***	**1**	**2**	**3**	**4**	**5**	**6**	**7**	**8**	**9**	**10**	**11**	**12**	**13**	**14**
1.	Resilience	334	20.53	4.99	-													
2.	AIMS: Overall	160	3.83	1.46	0.152	-												
3.	Social identity	160	3.73	1.51	0.192^*^	0.915^**^	-											
4.	Exclusivity	160	3.61	1.70	0.176^*^	0.879^**^	0.725^**^	-										
5.	Negative affectivity	160	4.21	1.82	0.022	0.856^**^	0.654^**^	0.638^**^	-									
6.	Overall wellbeing	349	44.03	14.31	0.516^**^	−0.053	0.010	−0.040	−0.123	-								
7.	Emotional wellbeing	349	10.77	3.08	0.517^**^	0.080	0.118	0.081	0.001	0.844^**^	-							
8.	Social wellbeing	349	13.21	5.94	0.389^**^	−0.107	−0.061	−0.073	−0.156	0.906^**^	0.660^**^	-						
9.	Psychological wellbeing	349	20.05	6.73	0.519^**^	−0.060	0.018	−0.061	−0.132	0.941^**^	0.755^**^	0.742^**^	-					
10.	Anxiety	339	7.50	4.35	−0.589^**^	−0.112	−0.161	−0.101	−0.020	−0.554^**^	−0.602^**^	−0.430^**^	−0.522^**^	-				
11.	Depression	339	5.13	3.87	−0.532^**^	−0.162	−0.227^**^	−0.109	−0.069	−0.682^**^	−0.714^**^	−0.555^**^	−0.632^**^	0.645^**^	-			
12.	Overall loneliness	329	2.62	0.75	−0.497^**^	−0.070	−0.149	−0.051	0.037	−0.635^**^	−0.639^**^	−0.524^**^	−0.594^**^	0.649^**^	0.709^**^	-		
13.	Emotional loneliness	329	2.88	0.80	−0.508^**^	−0.077	−0.139	−0.103	0.052	−0.612^**^	−0.649^**^	−0.482^**^	−0.578^**^	0.686^**^	0.668^**^	0.848^**^	-	
14.	Social loneliness	329	2.36	0.93	−0.368^**^	−0.050	−0.129	0.005	0.017	−0.502^**^	−0.477^**^	−0.433^**^	−0.465^**^	0.461^**^	0.573^**^	0.890^**^	0.513^**^	-

* *Correlation significant at the 0.05 level (2-tailed)*.

** *Correlation significant at the 0.01 level (2-tailed)*.

### Testing Hypothesis 2: Athletic Identity Will Be Negatively Correlated With Mental Health

All athletes and a subgroup of non-athletes (*n* = 160) were presented the AIMS. Whilst there was no correlation between non-athletes' AIMS-O and their mental health, athletes' AIMS-O a weak negative correlation with emotional wellbeing and weak positive correlations with HADS-A and each SLS variable which supports our second hypothesis. Each subfactor of AIMS was also significantly related to different aspects of mental health and illness (see Table 2 for descriptive statistics). Interestingly, AIMS-SI had a conflicting relationship with mental health and illness compared to the other AIMS variables. This meant that where significant relationships existed, increased AIMS-SI was related to better mental health whereas increased AIMS-O, AIMS-E and AIMS-NA scores were related to poorer mental health. A weak negative correlation also emerged between non-athletes' AIMS-SI and HADS-D: *r*(140) = −0.23, *p* = 0.007.

**Figure 4:**
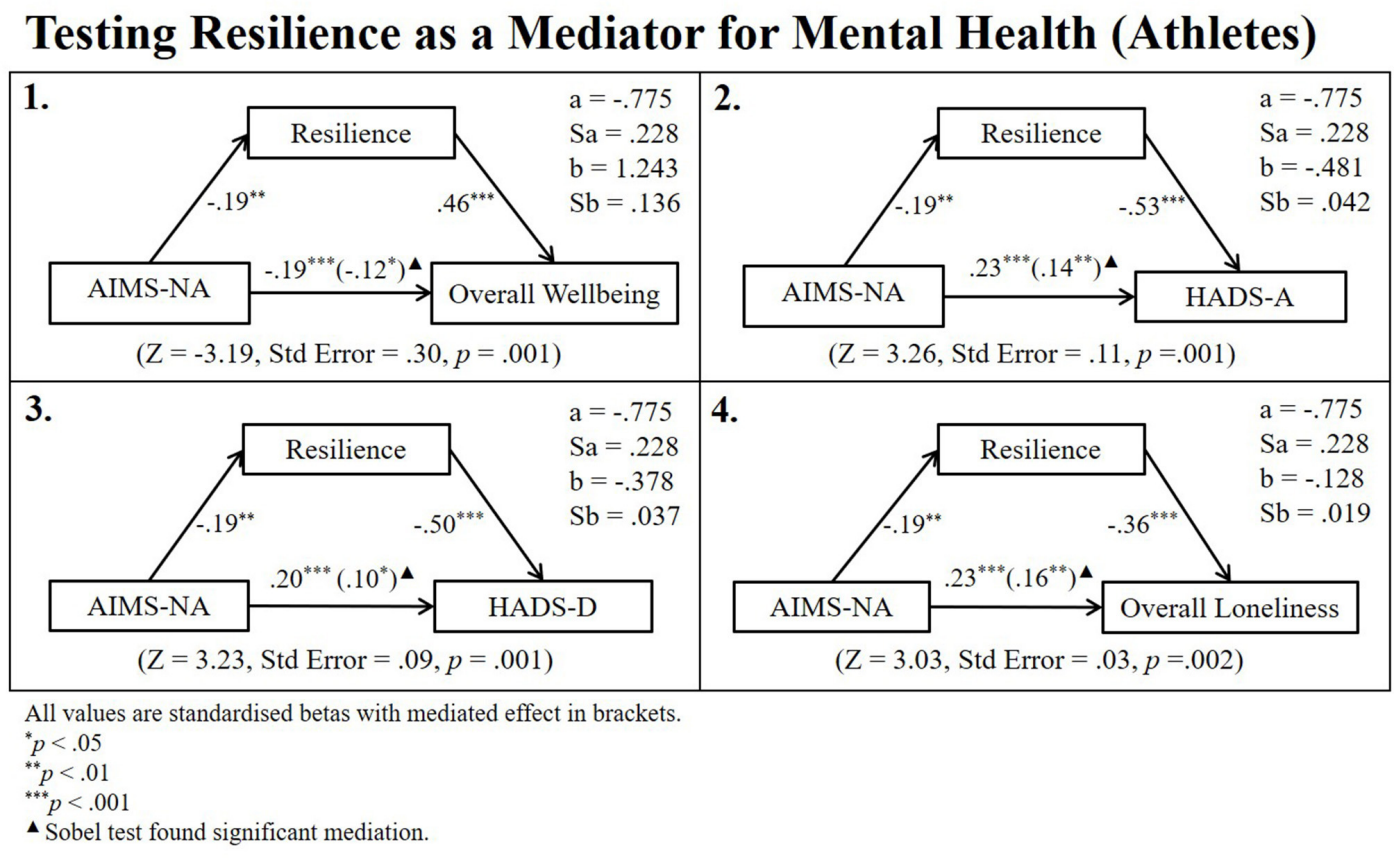
Regression path diagram testing resilience as a mediator for athlete sample.

**Figure 5:**
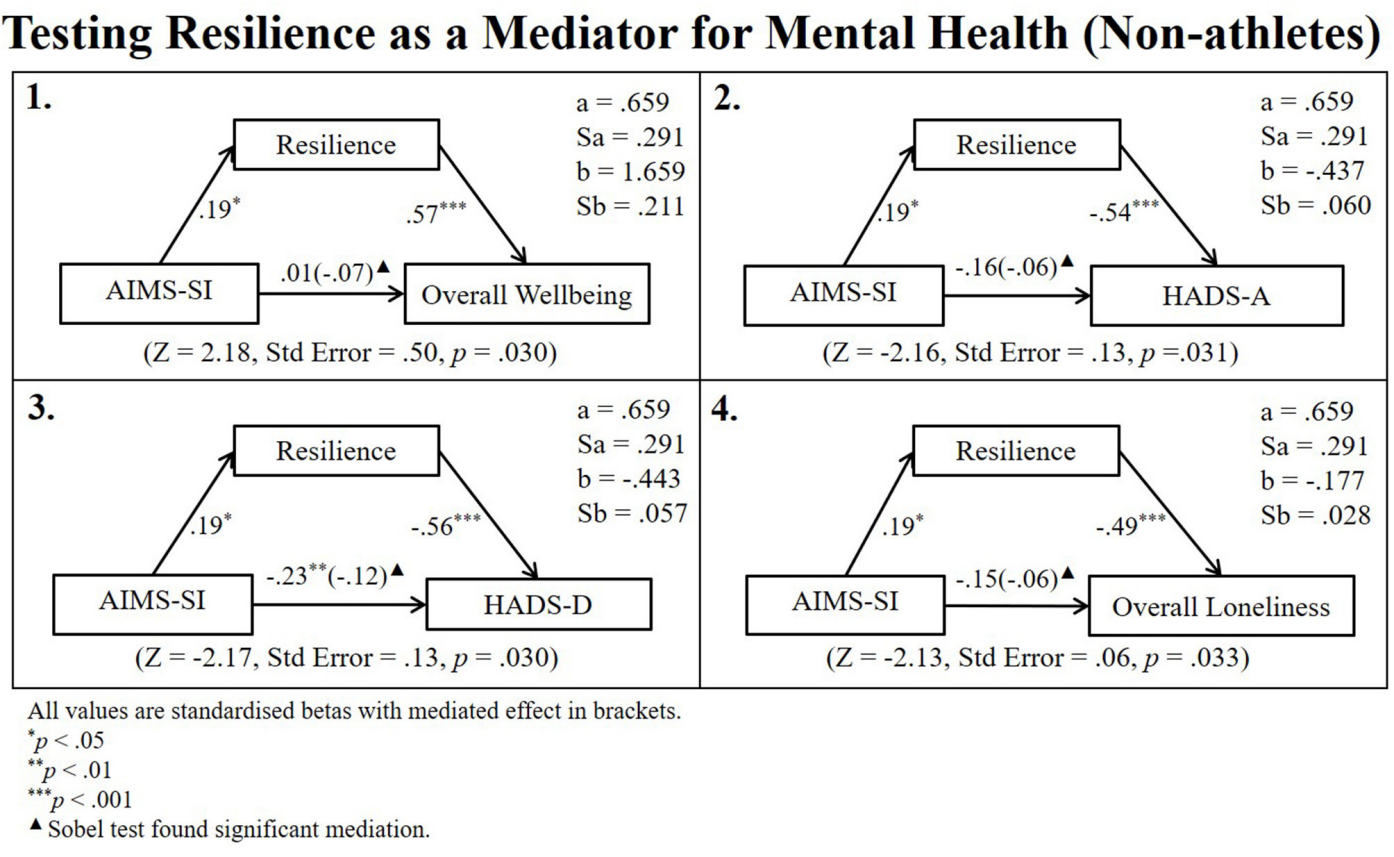
Regression path diagram testing resilience as a mediator for non-athlete sample.

Pearson's correlations revealed that athletes' AIMS-NA and non-athletes AIMS-SI scores were related to both resilience and mental health. Therefore, a series of linear regressions (Newsom, 2020) and subsequent Sobel tests for mediation were conducted to identify whether the relationship between aspects of athletic identity and mental health remained after controlling for potential mediation from resilience (see Figures 4, 5 for regression path diagrams). Sobel tests revealed that resilience partially mediated the relationship between athletes AIMS-NA and overall wellbeing (*z* = −3.19, std. error = 0.30, *p* = 0.001), HADS-A (*z* = 3.26, std. error = 0.11, *p* = 0.001), HADS-D (*z* = 3.23, std. error = 0.09, *p* = 0.001), and Lone-O (*z* = 3.03, std. error = 0.03, *p* = 0.002) and fully mediated the relationship between non-athletes' AIMS-SI scores and their HADS-D (*z* = −2.17, std. error = 0.13, *p* = 0.030).

### Testing Hypothesis 3: Resilience Will Be Higher in Athletes than Non-athletes

A two-way ANOVA was conducted to explore potential differences between males and females, athletes and non-athletes. No significant interaction was observed between the independent variables nor was there a difference between athletes and non-athletes. However, a gender difference emerged with males having greater resilience (*M* = 21.42, *n* = 301, *SD* = 4.89) than females (*M* = 20.48, *n* = 352, *SD* = 4.87): [F_(1, 649)_ = 4.37, *p* = 0.037, partial η^2^ = 0.007].

### Athlete and Non-athlete Differences

Independent sample's *t*-tests were conducted to test differences in athletic identity between athletes and non-athletes. As expected, there was a large effect with athletes scoring significantly higher than non-athletes on AIMS-O [*t*_(206.39)_ = 13.34, *d* = 1.63, *p* < 0.001 two-tailed]; AIMS-SI [*t*_(210.36)_ = 15.60, *d* = 1.92, *p* < 0.001 two-tailed]; AIMS-E [*t*_(259.77)_ = 9.15, *d* = 1.15, *p* < 0.001 two-tailed]; and AIMS-NA [*t*_(219.16)_ = 8.78, *d* = 1.17, *p* < 0.001 two-tailed]. These results survived Bonferroni adjustment for multiple comparisons (adjusted alpha level = 0.013).

**Figure 6:**
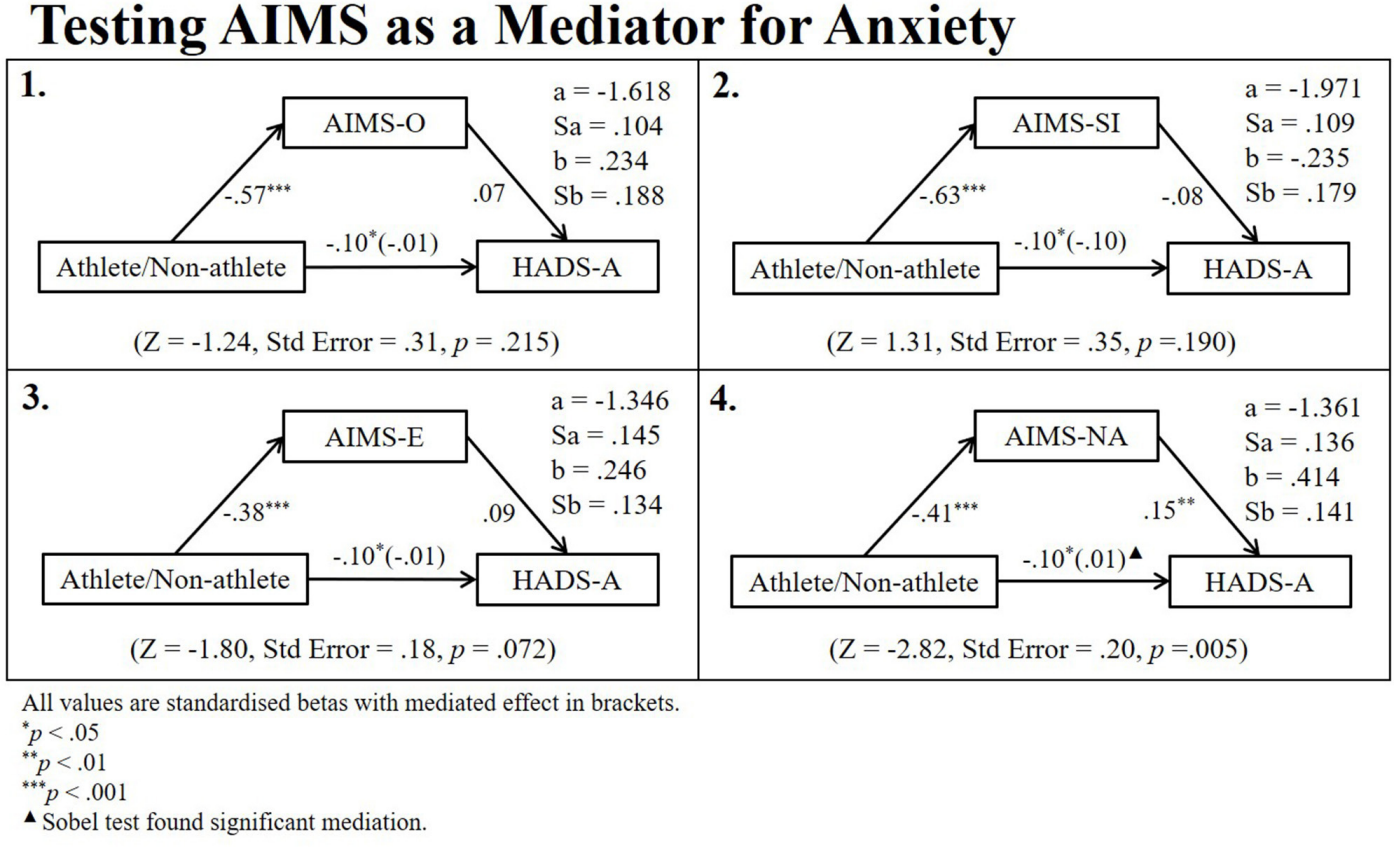
Regression path diagrams testing aims as mediators for the difference in athlete and non-athlete anxiety.

Two-way ANOVAs reported no differences in mental health other than for HADS-A where there was an effect of group (athlete/non-athlete): [*F*_(1, 659)_ = 6.05, *p* = 0.014, partial η^2^ = 0.009]; gender: [*F*_(1, 659)_ = 15.16, *p* < 0.001, partial η^2^ = 0.022]; but no interaction between the two: [*F*_(1, 659)_ = 0.09, *p* = 0.767]. Athletes (*M* = 8.10, *n* = 324, *SD* = 4.31) were more anxious than non-athletes (*M* = 7.50, *n* = 339, *SD* = 4.35) and females (*M* = 8.32, *n* = 359, *SD* = 4.35) were more anxious than males (*M* = 7.15, *n* = 304, *SD* = 4.25). A final series of regressions were conducted to test whether the difference in athlete and non-athlete HADS-A was mediated by AIMS. Figure 6 depicts the regression path diagram for these relationships. After controlling for the confounding effect of gender, there was no evidence that AIMS-O, AIMS-SI or AIMS-E mediated the difference in athlete and non-athlete HADS-A. However, the difference in athlete and non-athlete HADS-A went from significant to non-significant when AIMS-NA was added to the model. A significant Sobel test confirmed this was evidence of complete mediation by AIMS-NA (z = −2.82, Std Error = 0.20, p = 0.005).

## Discussion

The current study investigated resilience and athletic identity to improve our understanding of athlete mental health as we emerged from a COVID-19 lockdown. The findings support the first hypothesis—resilience would be positively correlated with mental health. For athletes, overall athletic identity was weakly positively correlated with anxiety and loneliness but had no relationship with wellbeing or depression, which partially supports the second hypothesis. Finally, there was no difference observed in the resilience levels of athletes and non-athletes, meaning the third null hypothesis was not rejected.

Until now, there was a dearth of research on athlete resilience during non-normative transitions. Existing studies of athlete resilience have used samples of athletes who finished training shortly prior to data collection or who otherwise had regular and ongoing involvement in sport (Guillén and Laborde, 2014; Laborde et al., 2016). As such, the resilience reported by these athletes may not have been representative of those going through a transition away from sport. We present evidence that during transitionary times such as COVID-19, there is no significant difference between the resilience of athletes and non-athletes. Instead, we propose that athlete resilience is circumstantial and may fluctuate depending on their current level of involvement in sport. The high prevalence of depression and substance abuse seen in athletes (particularly those performing at an elite level) during injuries or post-retirement would offer support to this claim (Alfermann et al., 2004; Appaneal et al., 2009). As well, the stigma toward mental health in sport and the sport-ethic demanding that athletes “put on a brave face” prevents some of those who are struggling from speaking out thus masking the true extent of mental illness in this population (Caddick and Ryall, 2012; Hilliard et al., 2020). We urge caution when advocating that sport participation develops resilience as there are evidently times when this is not the case.

The comparable levels of resilience between athletes and non-athletes were reflected in their mental health. Athletes reported similar levels of depression, loneliness and wellbeing as non-athletes. Alternatively, whilst males reported greater resilience and less anxiety than females, athletes had greater levels of anxiety than non-athletes despite having more males in the sample which signals that athlete specific stressors likely influenced their mental health. Specifically, 53% of athletes reported experiencing at least mild anxiety symptoms compared to 45% of non-athletes (see Table 1). These statistics are substantial enough to be a cause for concern for both athletes and non-athletes alike with social determinants likely contributing to why these figures are so high. The pressure of having to provide for children or care for those suffering from COVID-19 amid an unprecedented economic crisis has placed an incredible burden on the population. Concomitantly, job security has been drastically reduced with many individuals still facing the threat of redundancy. Indeed, when this survey was conducted, around 25% of the UK workforce were enrolled on the furlough scheme (Office for National Statistics, 2020)—an indication that job security was a wide-spread concern shared by athletes and non-athletes alike. However, these social pressures are not unique to athletes, and so cannot explain the significant difference in athlete and non-athlete anxiety. Instead, a psychological basis for the difference in athlete and non-athlete anxiety is more likely—a difference we attribute to their heightened athletic identity.

Research has shown that over-reliance on one's status as an athlete, can cause identity foreclosure (Murphy et al., 1996) and restrict the formation of a balanced and well-rounded sense-of-self causing some athletes psychological difficulties when they cannot compete in their sport. Our findings, depicted in Figure 6, provide evidence that after controlling for gender, negative affectivity—a subfactor of overall athletic identity—fully mediated the relationship between athletic status and anxiety. This finding infers that pandemic lockdown posed a significantly greater risk for athletes than non-athletes due to the high level of negative affectivity that comes with the athlete role. Alas, the cross-sectional nature of our study and lack of comparison data from before the lockdown limits the extent to which we can attribute these findings explicitly to the pandemic. To validate this evidence, future research should aim to follow athletes over time to try and capture athletic identity and mental health as athletes move from a period of uninhibited participation through a non-normative transition away from sport.

Another interesting finding was that whilst negative affectivity and exclusivity were associated with poor mental health, greater social identity was associated with more positive outcomes. Exclusivity and negative affectivity consider the degree to which an athlete's self-worth and emotional state are dependent on their ability to excel at sport (Brewer et al., 1993; Murphy et al., 1996). Moreover, an athlete's self-worth and their emotional state are more heavily influenced by the ability to play sport when they are intrinsically rather than extrinsically motivated to do so through social or societal pressures (Pelletier et al., 1995; Ryan and Deci, 2017). It is possible that athlete motives influence which aspects of athletic identity are developed thus causing different athletes to experience transitions differently. Research investigating the impact of motivation for athletic identity development would greatly enhance our understanding of athlete mental health and the risk factors for poor adaptation to transitions.

Our study comes with a number of limitations. First, we relied heavily on the use of self-report measures which in the past has led to under-reporting of disorders due to the stigma surrounding mental health in sport (Bauman, 2016; Hilliard et al., 2020). We mitigated this risk by preserving participant anonymity throughout making under-reporting of symptoms unlikely. Concurrently, collecting a large sample supports the likelihood that these findings offer an accurate and reliable representation of athlete mental health. Nevertheless, an additional (virtual) face-to-face interview would have improved the reliability of our findings. Second, it is possible that highly active non-athletes may have experienced some benefits of exercise for mental health and as such could have obscured significant differences between groups. Our ability to capture highly active individuals in the non-athlete group was limited as non-athletes were not asked how frequently they exercised. Having said that, these individuals were likely few in number and by strict definition, not athletes in sport and are therefore beyond our research questions. Finally, without pre-pandemic data for comparison, we cannot explicitly say that mental health has been significantly affected since the outbreak of COVID-19. Also, the dynamic nature of mental health means these statistics are liable to have changed in the months since the lockdown and will continue to change in perpetuity. Despite this, our findings make an important contribution to the field by “time-stamping” athlete mental health as the country emerged from an unprecedented national lockdown situation.

## Concluding Comments

This research contributes to the growing body of literature on sport participation and mental health, with the novel addition of capturing demographic and psychological factors during emergence from a national lockdown. Contrary to existing literature, athletes did not have greater resilience than non-athletes, possibly explained by the complex and likely uncomplimentary role that a narrow athletic identity with high negative affectivity serves during transitionary periods. Given social distancing measures are being intermittently eased and reinstated, the fate of both professional and amateur sport still hangs in the balance. Our findings have shown that it is now particularly important for research to continue to monitor and support the mental health of athletes.

## Equations

Cohen's *d* was calculated using the following formula (Howell, 2002):

$$\begin{array}{l} d=\frac{\left(M_{1}-M_{2}\right)}{square\;root\;of\;the\;pooled\;variance\;\left(sprv\right)} \end{array}$$

The square root of pooled variance was calculated using:

$$\begin{array}{l} sprv=\sqrt{\frac{\left(n_{1}-1\right){SD}_{1}+\left(n_{2}-1\right){SD}_{2}}{n_{1}+n_{2}-2}} \end{array}$$

Sobel tests used the following formula presented by MacKinnon and Dwyer (1993) where *a* = raw, unstandardised regression co-efficient for the relationship between the IV and mediator; *Sa* = standard error of *a; b* = raw coefficient for the relationship between mediator and DV when controlling for all other IVs; *Sb* = Standard error of *b*:

$$\begin{array}{l} z\;value\;=\;\frac{a\times b}{SQRT\left(b^{2}\times {s_{a}}^{2}+\;a^{2}\times {s_{b}}^{2}\right)} \end{array}$$

## Data Availability Statement

The data has been made available on Figshare online repository. The following DOI can be used to access the data: https://doi.org/10.6084/m9.figshare.14501361.v1.

## Ethics Statement

The studies involving human participants were reviewed and approved by Ulster University Research Ethics Filter Committee. The patients/participants provided their written informed consent to participate in this study.

## Author Contributions

CK, GB, and SS conceptualised the study with the same authors conducting early pilot work and data collection. CK processed the data. Analysis was conducted by CK and GP. All authors contributed to writing the paper.

## Conflict of Interest

The authors declare that the research was conducted in the absence of any commercial or financial relationships that could be construed as a potential conflict of interest.
